# The Regulation and Modification of GSDMD Signaling in Diseases

**DOI:** 10.3389/fimmu.2022.893912

**Published:** 2022-06-14

**Authors:** Zihao Li, Senlin Ji, Mei-Ling Jiang, Yun Xu, Cun-Jin Zhang

**Affiliations:** ^1^ Department of Neurology of Nanjing Drum Tower Hospital, Medical School and the State Key Laboratory of Pharmaceutical Biotechnology, Translational Medicine Institute of Brain Disorders, Nanjing University, Nanjing, China; ^2^ Institute of Brain Sciences, Nanjing University, Nanjing, China; ^3^ Jiangsu Key Laboratory for Molecular Medicine, Medical School of Nanjing University, Nanjing, China; ^4^ Jiangsu Province Stroke Center for Diagnosis and Therapy, Nanjing, China; ^5^ Nanjing Neuropsychiatry Clinic Medical Center, Nanjing, China

**Keywords:** GSDMD, signaling, regulation, diseases, therapy

## Abstract

Gasdermin D (GSDMD) serves as a key executor to trigger pyroptosis and is emerging as an attractive checkpoint in host defense, inflammatory, autoimmune diseases, and many other systemic diseases. Although canonical and non-canonical inflammasome-mediated classic GSDMD cleavage, GSDMD-NT migration to cell membrane, GSDMD-NT oligomerization, and pore forming have been well recognized, a few unique features of GSDMD in specific condition beyond its classic function, including non-lytic function of GSDMD, the modification and regulating mechanism of GSDMD signaling have also come to great attention and played a crucial role in biological processes and diseases. In the current review, we emphasized the GSDMD protein expression, stabilization, modification, activation, pore formation, and repair during pyroptosis, especially the regulation and modification of GSDMD signaling, such as GSDMD complex in polyubiquitination and non-pyroptosis release of IL-1β, ADP-riboxanation, NINJ1 in pore forming, GSDMD binding protein TRIM21, GSDMD succination, and Regulator-Rag-mTOR-ROS regulation of GSDMD. We also discussed the novel therapeutic strategies of targeting GSDMD and summarized recently identified inhibitors with great prospect.

## Introduction

Gasdermin-induced necrotic cell death called pyroptosis has been defined as a new type of cell death (1). Initially, pyroptosis was regarded as caspase-1–dependent necrosis for a long time to describe proinflammatory programmed cell death (2). As time passed and research advanced, especially with the discovery of inflammasome and the inflammasome pathway, pyroptosis was redefined as inflammasome-related cell death (3, 4). Furthermore, as the gasdermin protein family was identified to play a significant role in pyroptosis, particularly gasdermin D (GSDMD) serves as the key executor, the definition of pyroptosis was further integrated (5–8). In response to certain inflammatory signals, a diversity of inflammatory caspases like caspase-1, 4, 5 (in humans) and caspase-11 (in mice) are activated (6). Subsequently, with the disruption of plasma membrane integrity, tremendous inflammatory factors, such as Interleukin-1β (IL-1β), Interleukin-18 (IL-18), and tumor necrosis factor α (TNFα) were released (3, 9). Thus, pyroptosis is a unique cell death that has special mechanism and pathways.

There is no denying that GSDMD is an indispensable molecule in pyroptosis. GSDMD (also called GSDMDC1, DFNA5L, or FKSG10) was initially found in the homologues of GSDMA (10). Saeki et al. showed that GSDMD was widely expressed in different tissues and immune cells, including intestinal epithelial cells (IECs) and different subsets of leukocytes (11, 12). In terms of structure, GSDMD consists of an N-terminal domain (NTD, containing 242 amino acids) and a C-terminal domain (CTD, containing a 43–amino acid linker and 199 amino acids) (13). Notably, the GSDMD-NTD can induce pyroptosis by pore formation, whereas the expression of GSDMD-CTD is able to protect cells from pyroptosis (5). In normal status, GSDMD-NTD is in a special state called intramolecular autoinhibition by binding with GSDMD-CTD (5, 7). However, as long as the inflammatory pathway is activated and inflammatory caspases cleave the GSDMD to disrupt the autoinhibition (5, 6, 14, 15), the oligomerization of the GSDMD-NTD will occur (5, 6, 14, 16–18). Subsequently, numerous pyroptotic pores are formed, which are composed of GSDMD-NTD oligomers (5, 6, 19). Consequently, the GSDMD-NTD punch the cell membrane to form pores that can trigger pyroptosis and IL-1β release.

GSDMD also functions as the shared common effector of multiple inflammasomes that actively involved in a body of inflammatory and autoimmune diseases. Because of the critical function of GSDMD in inflammasome-related pyroptosis, GSDMD represents an ideal and novel target for therapeutic intervention. Early in 2006, Fink et al. demonstrated that pore formation of pyroptosis leads to release of caspase-1–activated cytokines from Salmonella-infected macrophages, suggesting pyroptosis as a novel pathway of inflammatory programmed cell death, whereas GSDMD-induced inflammatory pyroptosis was uncovered (20). Excessive or inappropriate pyroptosis can be highly pathological and lead to the development of numerous diseases (21). Conceivably, GSDMD acts as the crucial executor of pyroptosis and plays a significant role in various diseases, including autoimmune diseases, infectious inflammatory diseases, and other pyroptosis-associated diseases (22). GSDMD also has been confirmed as a key regulator and a therapeutic target in several diseases, like multiple sclerosis (MS), experimental autoimmune encephalomyelitis (EAE) (23, 24), cryopyrin-associated periodic syndromes (CAPS) (25–27), and Gram-negative bacteria–induced sepsis (6, 28, 29). Notably, the function and effect of GSDMD on other pyroptosis-associated diseases require closer study, such as Familial Mediterranean fever (FMF) (30–32), gout (33), Alzheimer’s disease (AD) (34), and Ischemic brain injury (35–37). Thus, carrying out the research of prohibiting pyroptosis *via* GSDMD signaling inhibitor has profound implications.

Up to date, the strategies of inhibiting GSDMD directly to repress pyroptosis in diseases have been evidenced. The mechanistic strategies include preventing the cleavage of GSDMD, as well as the oligomerization of GSDMD-NT fragments to reduce the formation of pyroptotic pores. In recent studies, several small-molecule inhibitors have emerged, like necrosulfonamide (NSA) (19), LDC7559 (38), magnesium (Mg^2+^) (39), disulfiram (40), and succination of GSDMD (41). These inhibitors can impede the cleavage of GSDMD or block the oligomerization of GSDMD-NT to mitigate pyroptosis.

In this review, we summarized the current advances of GSDMD, including GSDMD protein expression, stabilization, modification, activation, pore formation, and repair during pyroptosis. In addition to the lytic and non-lytic function of GSDMD, we also described some recent findings, including GSDMD complex in polyubiquitination and non-pyroptosis release of IL-1β, ADP-riboxanation, NINJ1 in pore forming, GSDMD binding protein TRIM21, GSDMD succination, and Regulator-Rag-mTOR-ROS regulation of GSDMD. Finally, we summarized that GSDMD is a key regulator and a therapeutic target in several diseases.

## The Transcriptional Regulation of GSDMD Expression

At the transcription level, two functioned binding sites of NF-κB were reported in the GSDMD promoter, including 250- to 100-bp and 720- to 250-bp upstream of the initiation site (42). Furthermore, attenuation of nuclear factor κB (NF-κB)–related GSDMD upregulation in adipocytes by melatonin, leading to the inhibition of inflammasome-induced and GSDMD-mediated pyroptosis (42). Consistently, the IL-1β release was also reduced in adipocyte after nuclear factor κB (NF-κB) inhibition. In addition, the hypermethylation of the promoter region mediated by DNMT (DNA methyltransferases) results in the reduced expression of GSDMD in NK92 cells, and enhancement of DNMT-mediated methylation-silencing of GSDMD by dimethyl fumarate (DMF) or monomethyl fumarate (MMF) was found to inhibit pyroptosis and IL-1β release (43), suggesting hypermethylation of GSDMD promoter region may serve as a critical checkpoint to transcriptionally modify the production of GSDMD in pyroptosis.

**Figure 1:**
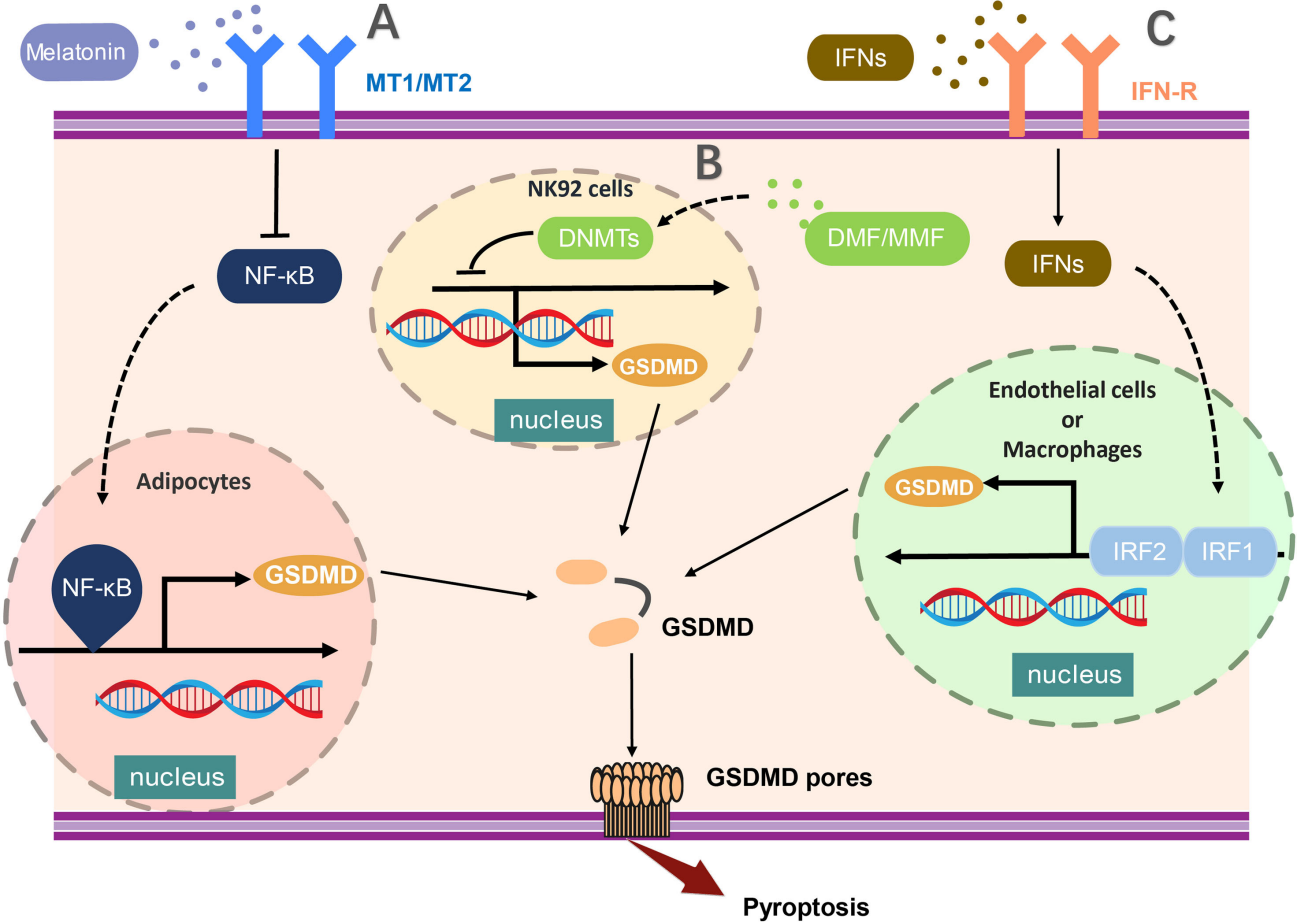
The transcriptional regulation of GSDMD expression. **(A)** NF-κB activation critically promotes the transcription of GSDMD in adipocytes and **(B)** the hypermethylation of the promoter region mediated by DNMT (DNA methyltransferases) results in the reduced expression of GSDMD in NK92 cells. **(C)** In endothelial cells or macrophages, GSDMD expression was profoundly enhanced by activation of both IRF1 and IRF2. The transcription of GSDMD could be regulated by multiple molecules, such as melatonin, DMF, and MMF. MT1/MT2, Melatonin receptor; NF-κB,nuclear factor κB; DNMT, DNA methyltransferases; DMF, dimethylfumarate; MMF, monomethylfumarate; IFNs, interferon; IRF1/2, Interferon Regulatory Factor 1/2.

Moreover, recent studies have demonstrated that interferon regulatory factor 2 (IRF2) is crucial to enhance the transcription of GSDMD for the execution of pyroptosis (44). IRF2 is one of the transcription factors of interferon regulatory factor family, which can bind to the specific site in GSDMD promoter leading to promotion of GSDMD transcription and expression (44). GSDMD expression was profoundly scaled down in *IRF2-*deficient endothelial cells and macrophages. Disruption of IRF2-binding site abolished the pyroptosis mediated by both the canonical and non-canonical inflammasomes significantly (44). Whereas, IRF1 was reported to regulate GSDMD gene expression in EA.hy926 cells in the absence of IRF2. It has been shown that the expression of GSDMD in *IRF2-*deficient cells could be rescued by IFNs induced IRF1 (44, 45). Interestingly, these regulation of GSDMD through IFNs and NF-κB was occurred in certain specific cells at the transcription level, due to the low stability of GSDMD in such cells. In conclusion, the regulation of GSDMD at the transcription level provided us a unique thought on pyroptosis suppression (**Figure 1**).

## The Lytic and Nonlytic Function of GSDMD

**Figure 2:**
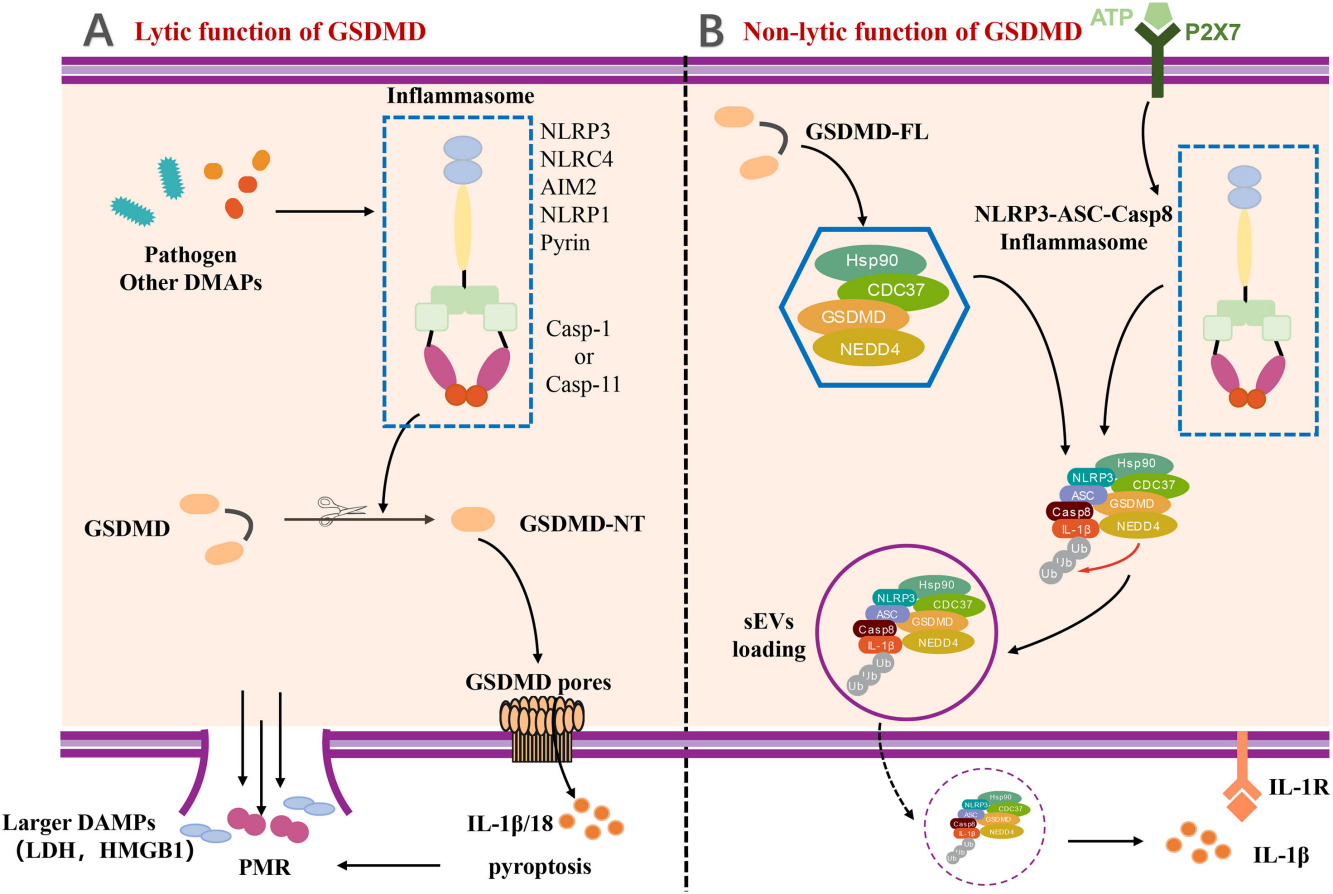
The lytic and non-lytic function of GSDMD. **(A)** The lytic function of GSDMD. Various inflammasome activated by pathogen and other DAMPs and further cleaved GSDMD to induce GSDMD-NT. With the oligomerization of GSDMD-NT, GSDMD pores formed and eventually resulted in plasma membrane rupture (PMR). IL-1β and other larger DAMPs released by lytic function of GSDMD. **(B)** The non-lytic function of GSDMD. A complex containing full-length GSDMD, chaperoned by the Hsp90 and CDC37, NEDD4 (an E3 ligase), and caspase-8, was formed in LPS-induced YAMC epithelial cells. The complex along with active caspase-8 and NEDD4 catalyzed polyubiquitination of pro–IL-1β induced release of IL-1β *via* sEVs by non-lytic function of GSDMD. DAMP, damage-associated molecular pattern; LDH, lactate dehydrogenase; HMGB1, high mobility group protein B1; PMR, Plasma membrane rupture; P2X7, P2X7 purinergic receptor; Hsp90, Heat Shock Protein 90; CDC37, cell division cycle protein 37; NEDD4, Neuronal precursor cell Expressed Developmentally Downregulated 4; sEVs, small extracellular vesicles.

GSDMD-mediated pyroptosis has been be classified as lytic cell death featured by membrane punching, cell swelling, and pore forming (**Figure 2**). In the pathway of canonical inflammasome-mediated GSDMD activation, certain pattern recognition receptors (PRRs), like Pyrin, AIM2, NAIP-NLRC4, NLRP3, and NLRP1, were activated after recognizing PAMPs and DAMPs and stimulation by second signaling agonist, which lead to the assemble of the canonical inflammasome (3, 7, 46). The formation of inflammsome requires the interaction between caspase recruitment domain PYD/CARD of different PRRs, the apoptosis-associated speck-like protein with a caspase recruitment domain (ASC) and pro–caspase-1 (7, 47). Pro–caspase-1 was auto-processed in the inflammasome to generate the activated caspase-1, which can cleave the proinflmmatory cytokines, such as pro–IL-1β and pro–IL-18, into bioactive forms (3, 48). Meanwhile, the autoinhibition state of GSDMD is also disrupted by activated caspase-1 to form the GSDMD-NTD (7). Subsequently, the GSDMD-NT migrates and interacts with the cell membrane by lipid binding site, followed by GSDMD-NT oligomerization, resulting in the pore forming, cell pyroptosis, and cytokine release. Moreover, the pores also lead to severe leakage of liposomes as well as dissolution of membranes including cellular membranes and organelle membranes (1, 5, 6, 49).

The GSDMD-mediated IL-1β release is generally considered to be taken place during pyroptosis. However, a few studies have reported that IL-1β can also be released dependent on a unique non-lytic function of GSDMD (50–52). The mechanism of non-lytic release of IL-1β has been determined in detail. IL-1β was first delivered to the extracellular space *via* aggregating in membrane vesicles like exosomes or secretory autophagy (53–56). It is noteworthy to mention that the Bulek et al. reported that the full-length GSDMD played an important role in the release of IL-1β within small extracellular vesicles (sEVs) from non-myeloid cells, such as IECs (53). Full-length GSDMD, along with a spectrum of novel GSDMD-interacting proteins, formed a new complex chaperoned by the Hsp90 and CDC37, NEDD4 (an E3 ligase), and caspase-8 in lipopolysaccharide (LPS)–induced young adult mice colonic (YAMC) epithelial cells. NEDD4 is known as an E3 ligase but plays a pivotal role in catalyzing polyubiquitination of pro–IL-1β. Along with the activation and assembly of caspase-8 inflammasome, NEDD4 catalyzes the polyubiquitination of pro–IL-1β and subsequently promotes the complex loading into secretory vesicles for IL-1β secretion (53), which provides a novel insight for us to understand the non-lytic function of GSDMD and inflammatory IL-1β secretion (**Figure 2**).

## The Canonical and Non-Canonical Pathway-Mediated GSDMD Activation in Pyroptosis

**Figure 3:**
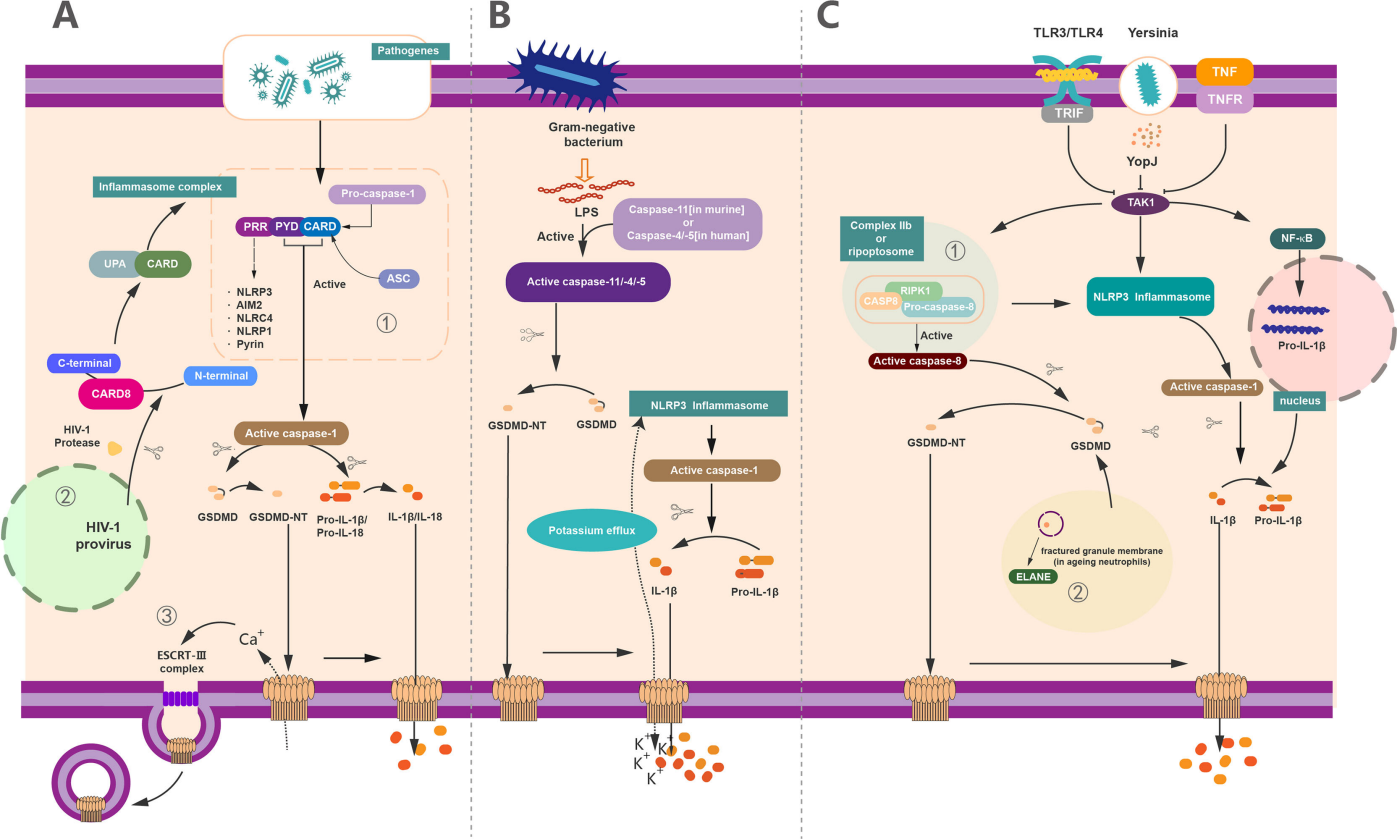
The regulation of GSDMD activation, pore forming and repair. **(A)** Canonical inflammasome pathway-mediated GSDMD activation in pyroptosis. (1) Canonical inflammasome pathway was activated upon recognition of exogenous and endogenous PAMPs, as well as DAMPs by PRRs. These PRRs recruit ASC and pro–caspase-1 *via* the interaction of PYD/CARD and further assemble these components together called inflammasome to generate activated caspase-1. Activated caspase-1 cleaved the full-length GSDMD and liberated the GSDMD-NT to insert into membrane and formed the pyroptotic pores. On the other hand, it can cleave pro–IL-1β/IL-18 into mature IL-1β/IL-18 and then release out of the cell together with cell content through GSDMD-pores to induce pyroptosis. (2) In addition to common PRRs, CARD8 can also act as a PRR to mediate the inflammatory process. HIV can be recognized by CARD8, causing pyroptosis. (3) 3Ca^2+^ influx can trigger the formation of ESCRT complex and initiate the membrane repair process. **(B)** Non-canonical inflammasome pathway-mediated GSDMD activation in pyroptosis. The activation of caspase-11 (in human) or caspase-4/5 (in murine) *via* lipopolysaccharide (LPS) produced by Gram-negative bacteria. These active caspases have the capacity to cleave GSDMD to GSDMD-NT and form membrane pores. Potassium efflux through membrane pores and further results in the assembly of NLRP3 inflammasomes. Similarly, whereas the active caspase-1 causes the maturation of IL-1β, these mature cytokines were released out of cell *via* the pyroptotic pores punched by oligomerized GSDMD-NT complex. **(C)** Other pathway-mediated GSDMD activation in pyroptosis and non-lytic function of GSDMD. (1) YopJ of Yersinia, TNF, or ligands of TLR3/TLR4 inhibit the inhibitory effect of TAK1. The elimination of the inhibitory effect of TAK1 leads to the activation of NLRP3 inflammasome and subsequently forms the active caspase-1 to cleave pro–IL-1β to IL-1β. The relief of TAK1 also induces the assembly of complex IIb (also called Ripoptosome), which contains RIPK1, FADD, and caspase-8. Active caspase-8 cleaves GSDMD into GSDMD-NT to form membrane pores which can release IL-1β. (2) In aging neutrophils, ELANE, produced from fractured granule membrane, induces the cleavage of GSDMD and further GSDMD-NT insert the membrane to form pores.

To cope with the infection of exogenous pathogens and certain endogenous damages, the immune system can protect the host by innate immunity and adaptive immunity. The innate immune system recognizes a set of pathogen-specific model molecules, called pathogen-associated molecular patterns (PAMPs) and damage-associated molecular patterns (DAMPs) (57, 58). These PAMPS and DAMPs include some structural components of bacteria, such as LPS, peptidoglycan (PGN), or nucleic acid molecules of viruses (57). Furthermore, the host possesses a battery of receptors to recognize these PAMPs, termed as PRRs (59). PRRs recognize a series of PAMPS during host defense and the occurrence of inflammation, which leads to the formation of inflammasome (4, 33, 60–64). The inflammasome is capable of activating a body of inflammatory caspases, containing caspase-1/4/5 in human and caspase-1/11 in mice (64, 65) (**Figure 3**). In the canonical inflammasome pathway, different inflammasomes are activated upon the stimulation of unique pathogens or non-pathogens (46). A host of studies have revealed that NLRP3 can recognize ATP or nigericin (9), NLRC4 can identify flagellin in the Salmonella enterica serovar Typhimuriumor (66, 67), AIM2 can identify the dsDNA ligand poly(dA:dT) (61, 68, 69), and Clostridium difficile toxin B can be recognized by Pyrin (30, 70). As reported, all of the above inflammasome could serve as the upstream of GSDMD and resulted in the cleavage of GSDMD by Casp to execute the pyroptosis. Notably, the activation of GSDMD could also feed-back control the activation of inflammasome. For example, potassium can be released from the GSDMD pores, causing the activation of NLRP3 inflammasome (7, 71), providing a new connection between canonical inflammasome and GSDMD activation (**Figure 3A**).

Unlike the canonical inflammasome pathway, the non-canonical inflammasome pathway leads to the activation of caspase-4/5 in humans and caspase-11 in mice (4, 72) (**Figure 3B**). LPS from Gram-negative bacteria can bind with these caspases and induce the activation and oligomerization of GSDMD without the involvement of inflammasome (63, 73–76). Likewise, the activated caspase-11 (caspase-4 and caspase-5 in humans) directly cleaves GSDMD and releases GSDMD-NTD, leading to the formation of membrane pores and pyroptosis (6, 15, 76). In addition to Casp-11, Mandal et al. showed that the collaboration between caspase-11 and caspase-8 supports the occurrence of endotoxic shock (77), supporting a critical role of Casp-8 in GSDMD-mediated pyroptosis. In analogy to canonical inflammasome pathway, activation of non-canonical inflammasome casp-11 pathway also results in the cleavage of pro–IL-1β/18 into mature IL-1β/18, which are subsequently secreted *via* GSDMD pores (**Figure 3B**).

## Other Pathway-Mediated GSDMD Activation

Recently, some other pathways of GSDMD-activating pyroptosis have been recognized beyond the canonical and non-canonical inflammasome pathway (**Figure 3C**). It has been demonstrated that Yersinia infection-induced inhibition of the transforming growth factor β–activated kinase (TAK1) or pharmacological inhibitor of TAK1 by 5Z-7-oxozeaenol (5z7) contributes to the cleavage of GSDMD without the participation of caspase-1/11 (78, 79). Mechanistically, GSDMD cleavage is directly driven by active caspase-8 *in vivo* and *in vitro* upon TAK1 inhibition (80). In detail, TAK1 strictly restrains the phosphorylation of receptor-interacting protein 1 (RIPK1) and the NLRP3 inflammasome (81, 82). In contrast, inhibition of TAK1 by TNF stimulation or Toll-like receptor 3/4 (TLR3 and TLR4) activation results in the release of RIPK1 (7, 78). The RIPK1 recruits Fas-associated *via* death domain (FADD) and pro–caspase-8 to assemble a complex called complex II b or Ripoptosome to activate the caspase-8 (7, 78, 79), which allow the activated caspase-8 subsequently to elicit the cleavage of GSDMD to induce pyroptosis (64). Meanwhile, the IL-1β is cleaved after NLRP3 inflammasome activation and secreted through the GSDMD membrane pores (83) (**Figure 3C**).

Except for the inflammatory caspases-related GSDMD activation, the neutrophil elastase (ELANE) in aging neutrophils has been recognized as an important molecule to cleavage GSDMD (84). In contrast to macrophage, where GSDMD-NT fragment was produced by the cleavage of a cascade of inflammatory caspases, the ELANE-dependent GSDMD activation and cleavage in neutrophils were caspase independent. Of note, ELANE-mediated GSDMD cleavage was suggested as the upstream of the caspase cleavage (85). The production of GSDMD cleavage mediated by ELANE in neutrophil is termed as ELANE-derived NT fragment (GSDMD-eNT), and it is capable of forming membrane pores to induce pyroptosis as caspase induced GSDMD-NT (85). In addition, the formation of neutrophil extracellular traps (NETs), a unique kind of neutrophil cell death that releases chromatin structures to the extracellular space, is reported to be correlated with GSDMD (38, 86). Particularly, ELANE-mediated cleavage of GSDMD promotes the formation of NETs in response to extracellular stimuli that trigger the activation of non-canonical inflammasome (64, 86). In addition, GSDMD-dependent death renders neutrophils produce antimicrobial NETs against cytosolic bacteria for defense (38, 86). All of these results suggest a distinct role of ELANE in GSDMD activation (**Figure 3C**).

## The Stabilization and Modification of GSDMD

In addition to the certain PRRs, like Pyrin, AIM2, NAIP-NLRC4, NLRP3, and NLRP1, the caspase recruitment domain-containing protein 8 (CARD8) also functions as a pathogen PRR to mediate the inflammatory process (87). CARD8 not only is associated with inflammasome-mediated pyroptosis in macrophages (88–90) but also recognizes all clinically isolated human immunodeficiency virus (HIV) subtypes to promote the pyroptosis in CD4^+^ T cells (87). In cells infected with HIV, the bioactive C-terminal CARD:caspase recruitment domain fragment of CARD8 is released for the assembly of inflammasomes, leading to GSDMD cleavage and GSDMD-mediated pyroptosis (87). As a critical mechanism of host defense, human immune system can recognize cells infected with HIV by CARD8 and then induces the activation of GSDMD-related pyroptosis to eliminate the latent virus and protects our body against AIDS (87) (**Figure 3**).

**Figure 4:**
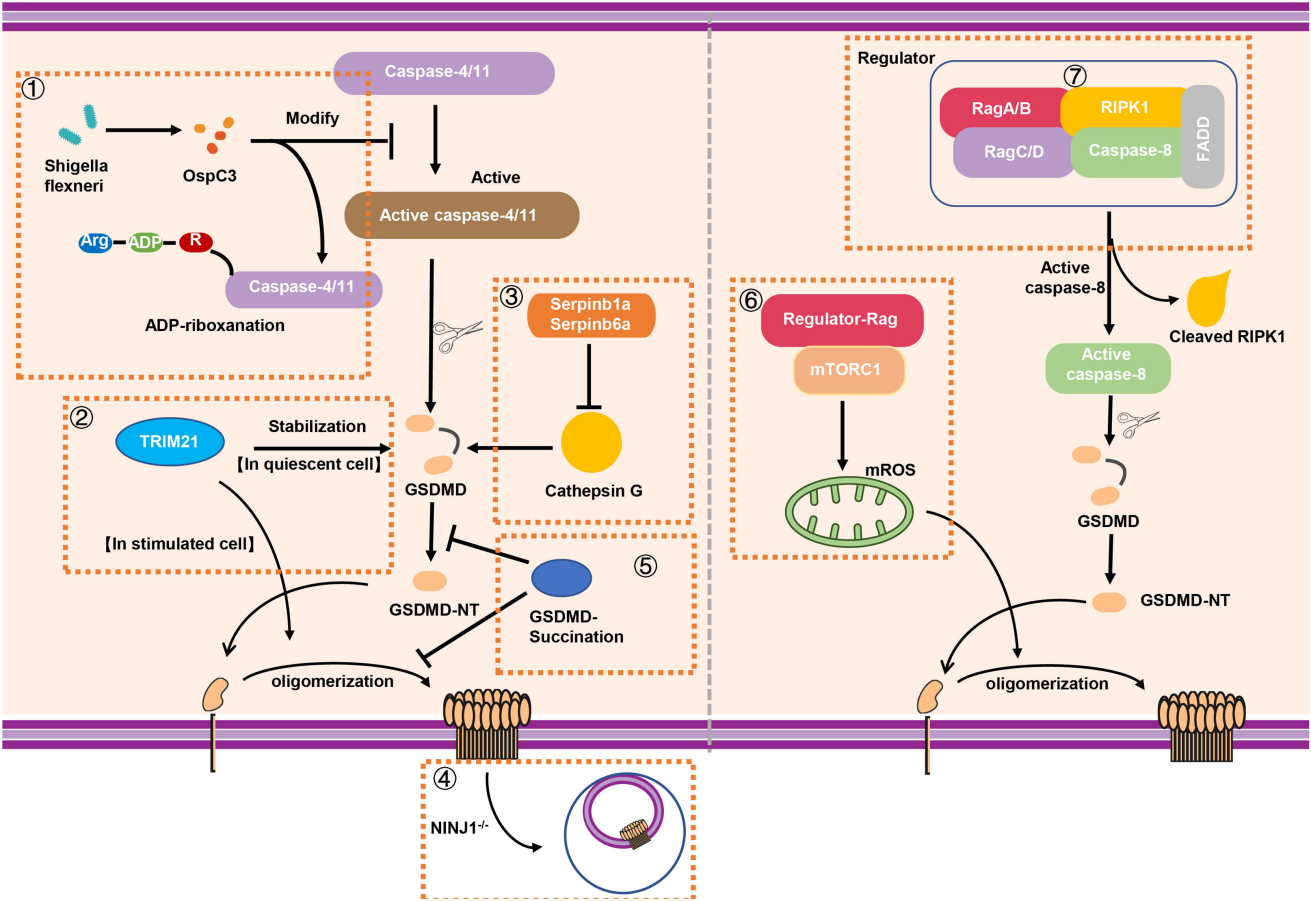
The stabilization and modification of GSDMD. (1) Shigella flexneri can secret the effector protein OspC3 to inhibit the function of caspase-4/11 *via* arginine ADP-riboxanation modification, leading to the inactivation of GSDMD signaling. (2) A novel binding protein of GSDMD called TRIM21 can stabilize GSDMD in quiescent cell while it promotes the oligomerization of GSDMD-NT for pyroptosis in stimulated cell. (3) In neutrophils, intracellular protease inhibitors Serpinb1a and Serpinb6a suppressed GSDMD-mediated neutrophil pyroptosis by inhibiting Cathepsin G (CATG) which promoting the cleavage of GSDMD. (4) The absence of NINJ1 in macrophage results in impaired plasma membrane rupture, but GSDMD remained the capacity of inducing the formation of membrane pores. (5) GSDMD succination not only prevents its interaction with caspases but also limits its processing, oligomerization and the ability to induce cell death. (6) Rag-Ragulator complex has the capacity to promote oligomerization of GSDMD in pyroptosis *via* mTORC1-ROS pathway. (7) Rag-Ragulator complex was involved in FADD-RIPK1-Caspase-8–mediated pyroptosis. RIPK1, Receptor-interacting protein kinase 1; FADD, Fas-associating protein with a novel death domain; mROS, mitochondrial reactive oxygen species.

On account of GSDMD that plays pivotal role in the occurrence of pyroptosis, the modification of GSDMD is growing evidenced. Humphries et al. discovered that GSDMD and GSDME can be modified with 2-(succinyl) on specific cysteine ​​sites by fumaric acid produced during metabolism (41). This succination modification of GSDMD inhibit the cleavage of full-length GSDMD as well as the oligomerization of GSDMD-NT to form membrane pores and prohibit the development of pyroptosis (41). GSDMD succination suppresses its interaction with caspases, which limits GSDMD processing, oligomerization, and the capacity to induce cell death, suggesting that GSDMD succination is an attractive strategy to restrain GSDMD activation (**Figure 4**). As early studies reported, 2-(succinyl)-cysteine ​​modification is an irreversible modification of DMF covalently to cysteine. Glyceraldehyde 3-phosphate dehydrogenase (GAPDH) and kelch like ECH associated protein 1 (KEAP1) has been confirmed can be modified by this (91, 92). Although it was demonstrated that Cys191 in human GSDMD (Cys192 in mouse) has the capacity of meditating GSDMD oligomerization (17), it can be modified with 2-succinyl in higher abundance (41). Of note, as a metabolic intermediate in the tricarboxylic acid cycle, fumaric acid demonstrated unique impacts on the modification of GSDMD and pyroptosis.

## Regulation of GSDMD Signaling in Pore Forming and Repair

The cell pyroptosis is featured by the presence of plasma membrane rupture (PMR), which is correlated with the production of damage-associated molecular patterns (DAMPs) and inflammatory response (93). As mentioned above, the process of pyroptosis requires the oligomerization of GSDMD-NT and the formation of the membrane pores, leading to the release IL-1β and LDH, the standard markers of the inflammation and PMR respectively. Ruan et al. found that LPS exposure promotes the release of a lectin called Galectin-1 *via* GSDMD-driven membrane pores and triggers inflammatory response (94). As reported, the size of GSDMD pores is about 21.5 nm inner diameter (95), which is much larger than IL-1β (about 4.5 nm diameter) and some large DAMPs. Although the importance of GSDMD in pore forming is known, the underlying mechanism of PMR is still to be determined. In 2021, Dixit et al. demonstrated that NINJ1 mediated PMR in lytic cell death (96). NINJ1, widely expresses in central nervous system (CNS), is served as an adhesion molecule related to inflammation and tumor inhibition (97). The absence of NINJ1 in macrophage results in impaired PMR and release of HMGB1 (a known DAMP) and LDH, supporting a dispensable role of NINJ1 in formation of membrane pores (96). In unstimulated BMDMs, NINJ1 exists in the form of dimer or trimer. However, upon the death stimuli, NINJ1 uses an evolutionarily conserved extracellular domain for self-oligomerization to induce PMR characterized by cell shrinkage and bleb formation (96). Interestingly, bleb formation is not prevented in NINJ1 deficiency BMDMs; however, a persistent ballooned morphology is developed to inhibit PMR (96). Although GSDMD is capable of inducing the formation of membrane pores, NINJ1 acts as a mediator of PMR on the other hand, supporting that cell death–related PMR is an active but not passive event (**Figure 4**).

Although the process of GSDMD cleavage, oligomerization, and pore forming has been well recognized, the mechanism of modification and regulating of GSDMD is still elusive. In 2021, Li et al. reported that Shigella flexneri infection can evade the occurrence of GSDMD-mediated cell pyroptosis (98). Interestingly, Shigella flexneri mediated a new type of post-translational modification through the secretion of the effector protein OspC3 to inhibit caspase-4/11. OspC3 interacts with caspase-4/11 to block its self-cleavage and activation induced by LPS. This process was dependent on a novel type of post-translational modification called arginine ADP-riboxanation. OspC3-modified caspase-4/11 lose the ability to bind and cleave GSDMD, leading to the escape of Shigella flexneri from the recognition and elimination during immune defense (98) (**Figure 4**). In addition, GSDMD can also be regulated by binding with other proteins, such as TRIM21. Gao et al. identified TRIM21 as a new regulator of GSDMD-mediated pyroptosis (99). TRIM21 directly interacts with GSDMD through the PRY-SPRY domain *in vivo* and *in vitro* to maintain the stable expression of GSDMD in cell (99). The discovery of this regulatory factor also provides a new target for fine-tuning and treatment of inflammation-related diseases. In addition, neutrophil serine proteases (NSPs), including cathepsin G (CATG), participated in GSDMD-associated immune response through a variety of ways (100, 101). Weinrauch et al. demonstrated that NSP is related to neutrophil death; however, the underlying mechanism has not yet been fully elucidated (102). In 2019, Burgener et al. found that while Cathepsin G cleaved GSDMD at Leu274 residue to generate a p30 fragment in neutrophil, the intracellular protease inhibitors Serpinb1a and Serpinb6a have the ability to prevent cathepsin G-dependent neutrophil death (103). In other words, Serpinb1a and Serpinb6a indirectly affect GSDMD-related neutrophil pyroptosis by inhibiting cathepsin G, supporting the important regulating role of these molecules in GSDMD activation in neutrophil (**Figure 4**).

It has been reported that pathogenic Yersinia was able to inhibit TAK1 to initiate Casp-8–triggered GSDMD cleavage and pore forming in macrophages; however, the regulator of GSDMD activation is not clear. The Evavold et al. established an experimental system to screen the factors of GSDMD-mediated membrane pore formation in the process of pyroptosis (104). Ragulator-Rag was discovered through genetic screening and identified as the key in the process of the formation of membrane pores. In 2021, two back-to-back studies reported Rag-Ragulator complex that functions as a novel key factor to regulate GSDMD-mediated pyroptosis (104, 105). Of note, this complex promotes the GSDMD activation independent of the canonical and non-canonical inflammasomes signaling. Specifically, the activity of mTOR (downstream of Ragulator-Rag) is indeed necessary for GSDMD-mediated pore formation. Notably, mTORC1, but not mTORC2, is participated in pyroptosis (104). While knockout of RagA or RagC does not affect the localization of GSDMD on the membrane, it does significantly impact the oligomerization of GSDMD (104). Furthermore, Zheng et al. explored the mechanism of Rag-Ragulator complex in FADD-RIPK1-Caspase-8–mediated pyroptosis (105). They found loss of Rag-Ragulator significantly inhibited a series of biological processes including the complexIIformation, RIPK1 phosphorylation, and caspase-8 activation, confirming that Rag-Ragulator complex (including RagA, RagC, and Lamtor1-5) actively participates in the regulation of Yersinia infection-induced GSDMD-mediated pyroptosis (105) (**Figure 4**).

Although activation of GSDMD results in the pore forming of cell membrane, pyroptosis does not necessarily to be the outcome of plasma membrane damage, because the repair programs like shedding as exosomes or the endocytosis of damaged membranes can be triggered by the influx of Ca^2+^ (106–109). Rühl et al. revealed that the endosomal sorting complexes required for transport **(**ESCRT) complex could be recruited to the damaged cell membrane during pyroptosis and subsequently initiates the repair process (52). ESCRT-III controls the integrity of plasma membrane and formed “bubbles” in plasma membrane disruption portions. With the shedding of “bubbles” from intact cells, the pores are repaired completely to preserve the common function of plasma membrane (110). In contrast, the inhibition of the ESCRT-III complex will remarkably aggravate the pyroptic cell death and IL-1β release in both human and mouse cells, whereas the cleavage of caspase-1 and GSDMD are grossly unaffected (52). In conclusion, ESCRT acts as a negative regulatory element of the downstream of GSDMD during pyroptosis and is able to repair the damaged plasma membrane (**Figure 3**).

## GSDMD Inhibitors and Mechanisms

### Necrosulfonamide

**Figure 5:**
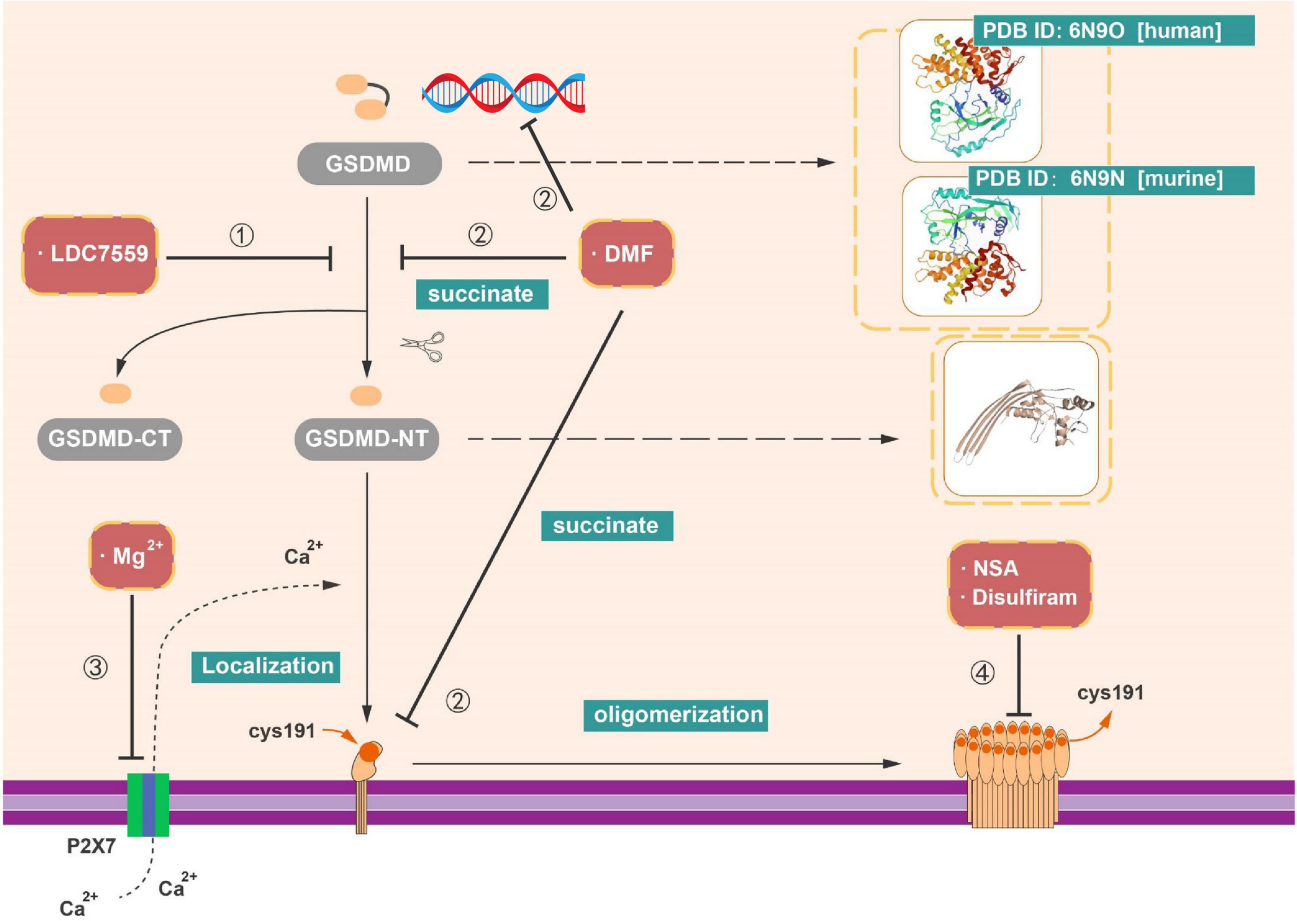
Inhibitors targeting on GSDMD. (1) LDC7559 can inhibit the cleavage of GSDMD as well as reduce the formation of GSDMD-NT. (2) DMF can regulate the transcription of GSDMD. In addition, with the succination of GSDMD, DMF can impede the cleavage and oligomerization of GSDMD. (3) Mg^2+^ is able to influence the function of GSDMD-NT, like the insertion of GSDMD-NT into membrane. (4) NSA, disulfiram, and Mg^2+^ have capacity to prohibit pore formation *via* inhibit the oligomerization of GSDMD-NT.

NSA was initially discovered *via* screening small-molecule inhibitors in the programmed necrosis (necroptosis) pathway in HT29 cells (111). NSA can combine with the mixed lineage kinase domain-like protein (MLKL) and destruct disulfide linkages in a cysteine at position 86 of human MLKL (111). Despite the fact that this pathway is different from pyroptosis, the disulfide bonds are involved in the oligomerization of GSDMD-NT (p30) and the formation of membrane pores (17). In 2018, Rathkey et al. demonstrated that NSA inhibited the aggregation of GSDMD-NT fragment in cells stimulated with LPS and nigericin by live confocal images of GSDMD-p30 fragment tagged with internal mNeonGreen in live cells (19). It revealed that NSA can target multiple inflammasomes, like NLRP3 and pyrin inflammasomes, to inhibit pyroptosis. Nevertheless, other innate immune or cell death pathways, such as TLR signaling and GSDME-meditated cell death, cannot be influenced by NSA (19). In the LPS-induced sepsis model, median survival increased obviously after the treatment with NSA. Collectively, NSA directly interacts with GSDMD and impacts the level of GSDMD-NT fragment oligomerization and subsequently reduces the formation of pyroptotic pores. Thus, NSA and optimized derivatives of NSA could be used in certain GSDMD-related inflammatory diseases (**Figure 5** and **Table 1**).

**Table 1:** GSDMD inhibitors and mechanisms.

Inhibitor	Year	Author	Function	Reference
**Necrosulfonamide (NSA)**	2012	Sun et al.	Inhibit necroptosis through binding to MLKL and disrupting disulfide bonds formed by Cys^86^ of human MLKL.	(111)
	2018	Rathkey et al.	Inhibit GSDMD-NT oligomerization by binding GSDMD directly at Cys^191^ (human GSDMD) and Cys^192^ (murine GSDMD).	(19)
**LDC7559**	2018	Sollberger et al.	Inhibit the cleavage of GSDMD in neutrophil cell death pathway (NETosis).	(38)
**Magnesium (Mg^2+^)**	2020	Wang et al.	Inhibit localization and oligomerization of GSDMD-NT *via* blocking the ATP-gated Ca^2+^ channel P2X7 to prevent Ca^2+^ influx.	(39)
**Disulfiram**	1990	Wright and Moore	Inhibit acetaldehyde accumulation of alcohol deterrent, which causes flushing and other aversive reactions.	(112)
	2017	Skrott et al.	Modification of NPL4, an adaptor of the p97 segregase, modulates multiple regulatory and stress-response pathways in cancer cells to promote their death.	(113)
	2020	Hu et al.	Inhibit GSDMD-NT oligomerization by targeting on Cys191 to reduce pore formation.	(40)
**Dimethyl fumarate (DMF)**	2018	Humphries et al.	Succinate and inactivate glyceraldehyde 3-phosphate dehydrogenase (GAPDH) at Cys^150^ and Cys^152^ in mice and humans.	(41)
	2020	Humphries et al.	Inhibit the cleavage of GSDMD by treating with GSDMD to form S-(2-succinyl)-cysteine at Cys^192^ cysteine residues (Cys^191^ in human).	(91)

### LDC7559

As previously described, GSDMD can act as an essential factor in the formation of NETs, an additional pathway of neutrophil cell death (NETosis) (38, 86). Nevertheless, it has become clear that a diversity of stimuli can induce NETosis, like phorbol 12­myristate 13­acetate (PMA) and reactive oxygen species (ROS) produced by reduced form of nicotinamide adenine dinucleotide phosphate (NADPH) oxidase complex (NOX2) (114–117). LDC7559 is a small molecule that efficiently inhibits PMA­induced NETosis (38). LDC7559 was found specifically binds to GSDMD *via* affinity chromatography and blocked the activity of GSDMD-NT to inhibit pyroptosis in murine immortalized bone marrow–derived macrophages (BMDMs), the THP­1, and human primary monocytes (38). In addition, LDC7559 also inhibited GSDMD cleavage and membrane integration in neutrophils (**Figure 5** and **Table 1**).

### Magnesium (Mg^2+^)

Hypomagnesemia is one of the clinically critical risk factors associated with poor outcome of sepsis patients (118). Recent research implied that magnesium has the capacity of protecting monocytes from death in sepsis (39). Notably, it is reported that the cleavage of GSDMD was not blocked by Mg^2+^, whereas the oligomerization and membrane localization of GSDMD-NT were limited not only in canonical but also in non-canonical pyroptotic pathway. As known, Mg^2+^ is an essential ion to sustain the health human body and serves as a powerful Ca^2+^ antagonist physiologically (119). In contrast to the effect of EGTA (a selective chelator of extracellular Ca^2+^) and BAPTA-AM (a strong chelator of intracellular Ca^2+^) in impeding pyroptosis, Mg^2+^ rescues GSDMD-induced diseases *via* blocking Ca^2+^ influx as indicated (39). Whereas Ca^2+^ influx is an instrument for the function of GSDMD-NT, Mg^2+^ blocks the ATP-gated Ca^2+^ channel P2X7, which plays a significant role in the membrane localization of GSDMD-NT during pyroptosis (39). Mg^2+^ is capable of alleviating diverse GSDMD-induced diseases in animal model, including sepsis in critically ill patients with hypomagnesemia, inflammation, and lung injury induced by LPS (39, 120). These findings provide the value of magnesium supplementation in sepsis prevention and treatment (**Figure 5** and **Table 1**).

### Disulfiram

Disulfiram is classically used as a drug for treatment of alcohol addiction in clinic (121), which inhibits acetaldehyde accumulation of alcohol deterrent and leads to flushing and other aversive reactions (112). Afterward, disulfiram is reported that it modulates multiple regulatory and stress-response pathways in cancer cell to promote cell death *via* modifying NPL4, an adaptor of the p97 segregase (113). Recently, Hu et al. performed a high-throughput screening and identified disulfiram as an inhibitor of GSDMD-induced liposome leakage to curb inflammation (40). They found that the effect of disulfiram on LPS-nigericin-induced IL-1β secretion in THP-1 and iBMDM is equivalent to the pan-caspase inhibitor z-VAD-fmk (40). Previous studies implied that disulfiram is rapidly metabolized to diethyldithiocarbomate (DTC), and complexation with copper gluconate [Cu(II)] would improve the activity of DTC (113, 122, 123). Supplement of Cu(II) was found to significantly enhance the protection of disulfiram against pyroptosis *in vivo* and *in vitro* (40). By the liposome leakage assay and negative staining electron microscopy, the disulfiram was suggested as a direct impediment drug of GSDMD pore formation. Moreover, the analysis of disulfiram-treated human GSDMD by using nano-liquid chromatography–tandem mass spectrometry (nano-LC–MS/MS) supported that Cys191 residue is of great important in GSDMD pore formation and can be targeted by disulfiram (40). These finding suggested the mechanism and function of disulfiram in suppression of GSDMD (**Figure 5** and **Table 1**).

### Dimethyl Fumarate

Modification of GSDMD by succination could prevent the interaction between GSDMD and caspases, restraining its cleavage, oligomerization, and block pyroptosis (41). Currently, DMF served as an oral immunosuppressive drug in disease-modifying therapies (DMTs) for the treatment of recurrent remission MS (RR-MS). DMF was mainly functioned through activating the nuclear transcription factor Nrf2. Whereas, in 2020, Humphries et al. found that DMF reduced the release of LDH and IL-1β in LPS plus Nigericin induced pyroptosis and blocked the formation of GSDMD-NT fragments. Several studies have confirmed that cell metabolism is connected to immune response. For instance, intermediates in Krebs cycle like itaconate and succinate both have impacts on inflammatory response (124–128). In contrast to DMF, monomethyl fumarate (MMF) failed to show any inhibitory effects (41). Mechanically, both exogenous treatments of DMF and endogenous accumulation of fumarate interacts with GSDMD to form S-(2-succinyl)-cysteine at Cys192 cysteine residues (Cys191 in human). Of note, succination of GSDMD mitigates its ability to interact with caspases and impedes its processing, oligomerization and pore formation (41). Moreover, DMF can also succinate and inactivate GAPDH at Cys150 and Cys152 in mice and humans (91). In line with other GSDMD-targeting drugs, succination of GSDMD offers a unique option for potential treatment of inflammatory diseases (**Figure 5** and **Table 1**).

### GSDMD and Autoimmune/Inflammatory Diseases

MS is recognized as a progressive autoimmune inflammatory demyelinating disease of the CNS. It has been shown that inflammasomes remarkably promote the initiation of inflammatory response in myeloid cells for disease progression (3). Whereas GSDMD could be the downstream of multiple inflammasomes, GSDMD-mediated pypoptosis is also observed in both myeloid cells (macrophages/microglia) and myelin-forming oligodendrocytes (ODCs) of MS and its animal model EAE (23). Consistently, another study revealed that the enhancement of activated GSDMD level was observed in the myeloid cell, as well as the surrounding blood vessels of EAE mice (24). FMF is the most prevalent monogenic autoinflammatory disease worldwide (31). FMF often carries a missense mutation of Mefv, leading to the overactivation of the Pyrin inflammasome and the downstream events, such as GSDMD-mediated pyroptosis and IL-1β secretion (30–32). In line with these results, Kanneganti et al. reported that GSDMD deficiency significantly protected against the FMF disease in murine model, including the improvement of anemia and growth retardation, the reduction of neutrophilia infiltration and inflammatory cytokine production (30). Another autoimmune disease related to NLRP3 mutations are known as CAPS, including familial cold autoinflammatory syndrome (FCAS), Muckle-Wells syndrome, and neonatal-onset multisystem inflammatory disease (NOMID) (129). Xiao et al. demonstrated that ablation of GSDMD, the potential downstream effector of NLRP3, dramatically prevented or ameliorated NOMID-induced inflammatory symptoms and disease progression (25–27), suggesting a potential clinical translational value of GSDMD in CAPS. In addition to NLRP3, mutation of NLRP1 also correlated with the occurrence of autoimmune disease. NLRP1-associated autoinflammation with arthritis and dyskeratosis (NAIAD) was known to be associated with NLRP1 mutations (130, 131). NLRP1 is a unique member of the NLR family and recent study has showed that chemical inhibitors of dipeptidyl peptidases (Dpp8 and 9) activate NLRP1b inflammasome in monocytes and macrophages (132). Mechanistically, DPP8 and DPP9 activate the pro–caspase-1 independent of the inflammasome adaptor ASC. In addition, activated pro–caspase-1 fails to efficiently process itself or IL-1β but cleaves gasdermin D to induce pyroptosis (133, 134), which highlights the important role of GSDMD in NAIAD (130) (**Table 2**).

**Table 2:** GSDMD and autoimmune/inflammatory/infection/metabolic and systemic diseases.

Disease	Year	Experimental System	Function and Mechanism of GSDMD in Disease	Reference
**MS**	2018 2019	Human microglia and ODCs, human brain tissue, GSDMD KO mice	* GSDMD-mediated pyroptosis in both myeloid cells (macrophages/microglia) and myelin-forming oligodendrocytes (ODCs). * GSDMD deficiency can result in the suppression of neuroinflammation and demyelination.	(23, 24)
**FMF**	2014 2017 2018	Gene knockout mice, Gene mutant mice (GSDMD KO mice)	* Missense mutations in *Mefv* activated the Pyrin inflammasome and GSDMD induced IL-1β secretion. * GSDMD-deficient would fully prevent runted growth, systemic inflammatory cytokine production, neutrophilia, and other characteristics in FMF.	(30–32)
**CAPS**	2013 2017 2018	Gsdmd KO mice, Nlrp3^L351PneoR^ and Nlrp3^A350VneoR^ mice	* Missense mutations in NLRP3 cause the disease to develop severe systemic inflammation driven by IL-1β and IL-18 overproduction as well as damage to multiple organs. * GSDMD can regulate the secretion of IL-1β and IL-18.	(25–27)
**NAIAD**	2017	Blood samples from patients, immortalized keratinocytes (N/TERT-1), PBMCs, gene knockout mice	The involvement of NLRP1 inflammasome associated with elevated systemic levels of caspase-1 and interleukin-18. GSDMD may serve as a treatment target in NAIAD.	(133, 134)
**Chronic proliferative dermatitis**	1996 2014 2016	Gene mutant mice (Sharpin^cpdm/cpdm^ mice), Gene knockout mice	Sharpin^cpdm^ mutation required components of the TNF-signaling pathway, NLRP3 inflammasome and IL-1R signaling to induce epithelial cell proliferation and multi-organ inflammation. GSDMD may participate in the disease and regulate the release of cytokines.	(135–138)
**RA**	2017 2015	N/A	IL-1, mainly IL-1β and Interleukin receptor antagonist (IL-Ra), plays crucial role in rheumatology. In line with it, GSDMD may adjust the expression of IL-1 to improve rheumatoid arthritis.	(139, 140)
**Gout**	2006 2019	Primary human monocyte and THP1, Mouse peritonitis model	The deposition of monosodium urate (MSU) or calcium pyrophosphate dihydrate (CPPD) crystals is involved in the caspase-1-activating NALP3 (also called cryopyrin) inflammasome and lead to the release of IL-1β and IL-18. GSDMD regulated the inflammatory cytokines release, and may become a potential therapeutic target.	(33, 141)
**Colitis**	2020	GSDMD KO mice, Gene knockout mice, colons from mice	GSDMD was activated during intestinal inflammation in the model of colitis and confirmed as a negative regulator controlling cyclic GMP-AMP synthase (cGAS)-dependent inflammation. In addition, GSDMD-mediated release of IL-1β *via* sEVs in the pathogenesis of colitis.	(53, 142)
**Bacterial infection (Sepsis)**	2015 2016 2018 2019	GSDMD KO mice, Gene knockout mice, THP1, HL-60, HeLa	LPS produced by Gram negative bacteria can mediate pyroptosis *via* non-canonical inflammasome pathway. The inhibition of GSDMD cleavage was confirmed as a key regulator and a treatment target in sepsis.	(6, 29, 32, 143, 144)
**Viral infection**	2013 2014 2017 2021	Human tonsil or splenic tissues, gene knockout mice	Quiescent lymphoid CD4 T cells die by caspase-1-mediated pyroptosis with the release of IL-1β. The role of Nod-Like-Receptor (NLR) during viral infection has been discovered, while the effect of GSDMD in this remains to be verified. Latest research elucidated that HIV-1 protease can induce the cleavage of CARD8 to activate the inflammasome formation and GSDMD-related pyroptosis.	(87, 145–149)
**Fungal infection**	2009 2015	Gene knockout mice	AIM2 and NLRP3 induce protective immune responses. The processing of IL-1β and IL-18 was controlled by Combined actions of caspase-1 and caspase-8. GSDMD may act as a novel part of the protective pathway to against Aspergillus infection.	(150, 151)
**Kidney diseases**	2008	IL-18^−/−^ mice, kidney IRI model	The release of IL-1β and IL-18 produced by macrophages was shown during ischemia-reperfusion injury (IRI) and many other renal cells death diseases. In a distinct setting, GSDMD participated in this progression and may be focused as immunogenicity and potential therapeutic interventions.	(152, 153)
**AD**	2019 2020	Mouse cortical neurons (Procell), transgenic mice (APP/PS1 mice and Tau22 mice), gene knockout mice	Aβ_1-42_ could induce pyroptosis in MCNs *via* GSDMD cleavage to increase membrane permeability and LDH release. The inhibitors targeting on GSDMD can lessen the Aβ_1-42_–induced pyroptosis and the behavioral ability in AD.	(34, 154)
**Ischemic brain injury**	2005 2019	Sprague–Dawley (SD) rats, MCAO/R model	The activation of NLRP1, NLRP3, NLRC4, and AIM2 inflammasomes were detected in the brain following ischemic stroke. Especially, the NLRC4 inflammasomes mediate the inflammatory response and pyroptosis in microglial cells, as well as the NLRP3 inflammasomes were assembled to increased levels of IL-1β and IL-18 in middle cerebral artery occlusion/reperfusion (MCAO/R). GSDMD participated in the progression of pyroptosis and may be a potential therapeutic target in ischemic brain injury.	(35–37)
**Cancer**	2017 2018	Human cervical cancer cell lines SiHa, ME-180, CaSki, SNU-17, and HeLa Clinical trials	In cervical cancer, the overexpression of Sirtuin 1 (SIRT1) is related to AIM2 inflammasome response. In addition, anti-inflammatory therapy targeting the IL-1β could significantly reduce lung cancer mortality. GSDMD served as an indispensable component of inflammasome pathway, may be a potential treatment target in several cancer.	(155–157)

MS, multiple sclerosis; EAE, experimental autoimmune encephalomyelitis; FMF, Familial Mediterranean fever; CAPS, cryopyrin-associated periodic syndromes; NAIAD, NLRP1- associated autoinflammation with arthritis and dyskeratosis; RA, rheumatoid arthritis; AD, Alzheimer’s disease.

*Separator between different reference studies.

Chronic proliferative dermatitis (CPD) is a disease featured by the presence of a mutation of Sharpin (Sharpin^cpdm^). The pathogenic characteristics of CPD have been confirmed to be correlated with the activation of RIPK1, MLKL, caspase-1/11, NLRP3, and IL-1β (135–138). The dermatitis in Sharpin^cpdm^ mice is analogous to the symptom in human suffering from CAPS, FMF, and neutrophilic dermatoses (158). NLRP3 inflammasome has also been demonstrated to be involved in dermatitis in Sharpin^cpdm^ mice (159). Gurung et al. highlighted a specific role of IL-1β, the downstream cytokine of NLRP3-GSDMD signaling, in provoking dermatitis in Sharpin^cpdm^ mice (138), suggesting that GSDMD may function as a potential checkpoint in CPD. Similarly, NLRP3 inflammasome also plays crucial role in promoting the development of rheumatoid arthritis (RA) and gout (33, 141). IL-1β and TNF are significantly increased in the serum of animal model of RA and play an important role in promoting the progression of RA (139, 140). In contrast, gout-associated uric acid crystals can activate NLRP3 inflammasome, resulting in GSDMD cleavage, IL-1β cytokine release, and the formation of membrane pores (33, 141), suggesting that GSDMD may be actively involved in these diseases. In addition, GSDMD is observed both in epithelial cell of patients with inflammatory bowel disease (IBD) and experimental colitis. In the murine model of colitis, GSDMD is activated during intestinal inflammation and is confirmed as a negative regulator controlling cyclic GMP-AMP synthase (cGAS)–dependent inflammation (142), providing a new clue to restrain colitis by regulating GSDMD (142). All these pieces of evidence support the critical function of GSDMD in a body of autoimmune or inflammatory diseases (**Table 2**).

## GSDMD and Infectious Diseases

For infectious diseases, LPS produced by Gram-negative bacteria can be delivered into the cytosol with outer membrane vesicles (OMVs) and trigger caspase-11–dependent pyroptosis (29, 143, 144). Consequently, several studies demonstrated that GSDMD is of great importance in LPS-induced sepsis (6, 28). Consistently, GSDMD deficiency protected mice from the lethality in LPS-induced sepsis (6) (**Table 2**).

In addition to sepsis caused by bacteria, certain viral infectious diseases are also tightly correlated with pyroptosis, such as HIV (160, 161). CD4^+^T cell intrinsic NLRP3–Caspase-1 mediates pyroptosis and drives the T cell depletion after HIV-1 infection, whereas the role of GSDMD in this process remains to be eliminated (161). Similar to HIV, both DNA virus and RNA virus infection, like influenza A virus and zika, lead to the formation of inflammasomes and the production of various inflammatory cytokines, including IL-1β (145, 146). However, the activation of canonical and non-canonical inflammasome block cGAS-dependent signaling and impacts the host defense during DNA virus infection (145). Enterovirus 71 (EV71), a virus that is capable to trigger hand-foot-and-mouth disease (HFMD), can elicit cleavage of GSDMD independent of caspase-1 (147). All of these studies might provide a crucial role of GSDMD prevention and treatment of viral infection diseases. In addition to bacteria and virus infection, the role of GSDMD has also been proposed during fungal infection. Indeed, caspase-1/11–induced poroptosis was initiated immediately after fungal infection, especially in immunocompromised patients (162). IL-1β has been proven as a crucial factor for the immune response to fungal infections (163). Several studies have shown that NLRP3-deficient mice are more susceptible to Candida albicans infection (150). Karki et al. revealed that Aspergillus fumigatus induce the assembly of AIM2 and NLRP3 inflammasome (151). Although the role of GSDMD in fungal infectious diseases has not been defined exactly, as the downstream of AIM2 and NLRP3 inflammasome, GSDMD is believed to play critical role in these diseases (**Table 2**).

## GSDMD and Other Organ Disorders

In certain kidney diseases of the urinary system, the renal cells are capable of triggering acute tubular necrosis, autoimmunity, ischemia-reperfusion injury (IRI), and necrotizing glomerulonephritis (152). Renal cell dysfunction often drives inflammasome-associated caspases to cleavage and release IL-1β or IL-18 *via* GSDMD-mediated plasma membrane pores (152, 153). Similar to renal cell, the neuron in brain could also be induced to pyroptosis. It has been demonstrated that β-amyloid was capable of inducing neuronal pyroptosis in AD (34, 154). Aβ_1-42_ exposure leads to the upregulation of GSDMD-NT (p30), which was cleaved by the NLRP3–caspase-1 inflammasome in mice cortical neurons (MCNs) (34). The pyroptosis of neuron also takes place in stroke, one of the Top2 causes of death and disability worldwide (164). In middle cerebral artery occlusion/reperfusion (MCAO/R) stroke model, the elevation of GSDMD, IL-1β, and IL-18 was observed (37). It is demonstrated that not only GSDMD (37) but also NLRC4 and NLRP3 have been identified to promote microglia or neuronal pyroptotic cell death in ischemic stroke (35, 36). Consistently, disruption of NLRC4 inflammasome reduced the production of IL-1β and IL-18 and attenuated the disease severity of cerebral ischemia injury (35). These results suggested that NLRC4-GSDMD signaling plays an important role in promoting the disease progression (**Table 2**). Whereas over-activated pryoptosis of the tissue cells results in the impairment of the normal function of our body, the dysregulation of pyroptosis is actively engaged the development and progression of various cancers, including cervical cancer, lung cancer, and gastrointestinal tumors (155–157). For example, the activation of NLRP6 and NLRP6-dependent pyroptosis and IL-18 secretion has been characterized to improve the antitumor immunity by maintaining a healthy gut microbiota (157). Elsewhere, the inhibition of IL-1β by canakinumab significantly reduces the incidence and mortality of lung cancer as observed in a clinical trial, suggesting that pyroptosis may play distinct role in different cancers (156). Despite the importance of pyroptosis has been recognized in cancers, the unique mechanism of GSDMD in tumor growth, invasiveness, and metastasis still needs future exploration (**Table 2**).

## Conclusion and Future Perspective

Because GSDMD was identified as the direct executor of pyroptosis, completely understanding the regulatory mechanisms of GSDMD, such as GSDMD protein expression, stabilization, modification, activation, pore formation, and repair during pyroptosis, is critical in the field of biological science and clinic. Although the GSDMD cleavage by classic inflammatory caspases like caspase-1/11/8 is well known, the neutrophil elastase (ELANE) can also cleave the full-length of GSDMD, suggesting that the mechanism of GSDMD cleavage may be cell specific. In addition, the knowledge on the outcome of cells with GSDMD activation has also been advanced. The GSDMD-NT could migrate to the cell membrane and oligomerization to form the GSDMD-NT complex, rendering the release of cytosolic contents and resulting in lytic pyroptosis. In contrast, the non-lytic function of GSDMD has been summarized, providing a unique insight of GSDMD on cell function and disease. Currently, almost all of the GSDMD-related literatures found IL-1β was released *via* the cell membrane pores in macrophage; however, the secretion of IL-1β in gut epithelial cell is dependent on the full-length GSDMD in colitis. Whether the later mechanism still take place in other cells is still to be eliminated.

Because GSDMD serves as the common co-effector of inflammasome, blockade of GSDMD by prohibition against the cleavage or oligomerization of GSDMD *via* certain small molecule has been proven to work in various diseases. So far, the currently available inhibitors of GSDMD (such as NAS, DMF, and Disulfiram) also impact the upstream signaling of GSDMD, such as NF-κB, casp-1 cleavage by modified reactive cysteines covalently. In-depth recognition of mechanisms of regulating pyroptosis and executive function of GSDMD will not only expand our current knowledge but also enable to develop novel and more specific inhibitors. Furthermore, despite of the fact that GSDMD is involved in numerous diseases, the inhibitors targeting GSDMD have not been successfully developed in clinic until now. Some clinical drugs, such as DMF and disulfiram, are well-tolerance and has the potential for clinical trials by conventional drug in new use. It is critical to further discover the more specific GSDMD inhibitors that may directly block GSDMD-NT activity to repress the pore formation. To this end, our group found that a small molecular C202-2729 specifically interacts with GSDMD and blocks its lipid-binding site without impacting on the activation of GSDMD upstream proteins. Although more selective GSDMD inhibitors are still required, the identification and optimization inhibitors will undoubtedly promote the clinical translation usage.

In regard to pore formation, emerging work presents a new mechanism about membrane repair by recruiting the endosomal sorting complexes required for transport (ESCRT) that can negatively regulate pyroptosis (52, 110). Some regulating mechanisms of GSDMD are also uncovered, such as GSDMD complex in polyubiquitination and non-lytic function of GSDMD in IL-1β release, ADP-riboxanation, NINJ1 in pore forming, GSDMD binding protein TRIM21, GSDMD succination, and Regulator-Rag-mTOR-ROS regulation of GSDMD. All of the above mechanism will be favorable for drug discovery and development. For example, as inspired by fumarate produced metabolites, promotion of the succination of GSDMD by DMF to repress GSDMD activation come to light (41). Other endogenous metabolite compounds like heparin are capable of blocking the transportation of LPS into the cytoplasm to prevent caspase-11–related septic lethality and pyroptosis (165). Endogenous metabolite compounds are also becoming attractive candidates to inhibit the GSDMD-dependent pyroptosis. In conclusion, although the regulation mechanism of GSDMD has achieved great advances, the unique pattern of GSDMD activation in distinct cell type or disease is still required further exploration. Despite multiple GSDMD inhibitors have been identified, more selective and tolerance inhibitors are still required for clinical translation. GSDMD is becoming a very attractive checkpoint in multiple diseases, and it is opening new window for therapeutic intervention against a body of GSDMD-involved diseases.

## Author Contributions

C-JZ and YX conceived the study. C-JZ, ZL, SJ, and MJ wrote the manuscript. All authors contributed to the article and approved the submitted version.

## Funding

This study was supported by the National Natural Science Fund for Excellent Young Scholars (82022019 to C-JZ), Nanjing special fund for Medical Science and Technology Development Projects for Distinguished Young Scholars (JQX19005 to C-JZ), and National Natural Science Foundation of China (82101414 to MJ, 81701235 to C-JZ, and 81991514 to C-JZ).

## Conflict of Interest

The authors declare that the research was conducted in the absence of any commercial or financial relationships that could be construed as a potential conflict of interest.

## Publisher’s Note

All claims expressed in this article are solely those of the authors and do not necessarily represent those of their affiliated organizations, or those of the publisher, the editors and the reviewers. Any product that may be evaluated in this article, or claim that may be made by its manufacturer, is not guaranteed or endorsed by the publisher.
